# Adaptive Change Inferred from Genomic Population Analysis of the ST93 Epidemic Clone of Community-Associated Methicillin-Resistant *Staphylococcus aureus*

**DOI:** 10.1093/gbe/evu022

**Published:** 2014-01-29

**Authors:** Timothy P. Stinear, Kathryn E. Holt, Kyra Chua, Justin Stepnell, Kellie L. Tuck, Geoffrey Coombs, Paul Francis Harrison, Torsten Seemann, Benjamin P. Howden

**Affiliations:** ^1^Department of Microbiology and Immunology, University of Melbourne, Victoria, Australia; ^2^Department of Microbiology, Monash University, Clayton, Victoria, Australia; ^3^Department of Biochemistry and Molecular Biology, Bio21 Institute, University of Melbourne, Victoria, Australia; ^4^Austin Centre for Infection Research (ACIR), Infectious Diseases Department, Austin Health, Heidelberg, Victoria, Australia; ^5^Department of Microbiology, Austin Health, Heidelberg, Victoria, Australia; ^6^School of Chemistry, Monash University, Clayton, Victoria, Australia; ^7^Australian Collaborating Centre for *Enterococcus* and *Staphylococcus* Species (ACCESS) Typing and Research, School of Biomedical Sciences, Curtin University, Bentley, Western Australia, Australia; ^8^Department of Microbiology and Infectious Diseases, PathWest Laboratory Medicine WA, Royal Perth Hospital, Perth, Western Australia, Australia; ^9^Victorian Bioinformatics Consortium, Monash University, Clayton, Victoria, 3800, Australia

**Keywords:** *Staphylococcus aureus*, community-acquired MRSA, comparative genomics, alpha-hemolysin

## Abstract

Community-associated methicillin-resistant *Staphylococcus aureus* (CA-MRSA) has emerged as a major public health problem around the world. In Australia, ST93-IV[2B] is the dominant CA-MRSA clone and displays significantly greater virulence than other *S. aureus*. Here, we have examined the evolution of ST93 via genomic analysis of 12 MSSA and 44 MRSA ST93 isolates, collected from around Australia over a 17-year period. Comparative analysis revealed a core genome of 2.6 Mb, sharing greater than 99.7% nucleotide identity. The accessory genome was 0.45 Mb and comprised additional mobile DNA elements, harboring resistance to erythromycin, trimethoprim, and tetracycline. Phylogenetic inference revealed a molecular clock and suggested that a single clone of methicillin susceptible, Panton-Valentine leukocidin (PVL) positive, ST93 *S. aureus* likely spread from North Western Australia in the early 1970s, acquiring methicillin resistance at least twice in the mid 1990s. We also explored associations between genotype and important MRSA phenotypes including oxacillin MIC and production of exotoxins (α-hemolysin [Hla], δ-hemolysin [Hld], PSMα3, and PVL). High-level expression of Hla is a signature feature of ST93 and reduced expression in eight isolates was readily explained by mutations in the *agr* locus. However, subtle but significant decreases in Hld were also noted over time that coincided with decreasing oxacillin resistance and were independent of *agr* mutations. The evolution of ST93 *S. aureus* is thus associated with a reduction in both exotoxin expression and oxacillin MIC, suggesting MRSA ST93 isolates are under pressure for adaptive change.

## Introduction

Methicillin-resistant *Staphylococcus aureus* (MRSA) strains are common throughout the world (Zinn et al. 2004) and until recently had been confined to hospitals. Over the past decade, the global emergence of community-acquired MRSA (CA-MRSA) has been a remarkable phenomenon, dominated by the rapid emergence and spread of a clone of CA-MRSA in the United States, called USA300 (Moran et al. 2006; Klevens et al. 2007; Kennedy et al. 2008). USA300-0114 is ST8, SCC*mec*-IV and carries the genes (*lukS-PV* and *lukF-PV*) encoding the Panton-Valentine leukocidin (PVL). Active case surveillance found an incidence of invasive CA-MRSA of 4.6 per 100,000 (Klevens et al. 2007). The USA300 clone has caused large numbers of severe SSTIs and necrotizing pneumonias in otherwise healthy individuals and is replacing “traditional” hospital clones of MRSA causing severe infections in hospitalized patients while continuing to cause serious infections in otherwise healthy individuals in the community (Seybold et al. 2006).

USA300 possesses other virulence factors that include the arginine catabolic mobile element that assists with bacterial survival and is postulated to play a role in the rapid clonal spread of USA300 and its apparent hypervirulence (Diep and Otto 2008). However, it is difficult to be certain of the individual contribution of any one factor, and comparative genomic and functional assessments suggest that the increased virulence of USA300 may be due to upregulation of core virulence genes such as Hla and phenol soluble modulins (Li et al. 2009).

In Australia, CA-MRSA first emerged in the remote Kimberley region of Western Australia (WA, Udo et al. 1993) with Aboriginal ethnicity as a major risk factor for CA-MRSA infection (Maguire et al. 1996). In the late 1990s, CA-MRSA infections were noted in Eastern Australia, especially affecting the Polynesian community (Nimmo et al. 2000; Gosbell et al. 2001). Recent results from the Australian Group on Antimicrobial Resistance (AGAR) community staphylococcal surveillance program (2012) demonstrated a significant rise in MRSA in Australia, entirely due to an increased prevalence of CA-MRSA, not hospital-associated MRSA (AGAR 2012). A large number of clones of CA-MRSA have been described in Australia, based on MLST and SCC*mec* typing (Nimmo et al. 2006; Nimmo and Coombs 2008; Coombs et al. 2009).

Over the past 6 years, a new clone of CA-MRSA that is rarely reported outside Australia and was first reported in Queensland (QLD, Munckhof et al. 2003), the ST93 QLD clone or ST93-QLD, has become the most common CA-MRSA clone throughout Australia (Coombs et al. 2006). In fact, the increase in MRSA infections documented in the last AGAR survey was due entirely to an increase in the prevalence of the ST93-QLD CA-MRSA, a situation reminiscent of the start of the USA300 epidemic in the United States. ST93-QLD carries the SCC*mec* type IV[2B] element and the *lukSF*-PV genes encoding PVL (Vandenesch et al. 2003). However, not all ST93 strains are alike. They are not all MRSA, and some ST93 are PVL negative. A study using *spa* gene high-resolution melt curve profiles confirmed some genetic diversity within ST93 (Tong et al. 2009).

ST93-QLD CA-MRSA is associated with severe clinical disease, and although it commonly causes significant skin and soft tissue infections, it has also been associated with fatal necrotizing pneumonia, bacteraemia secondary to skin infections, and deep musculoskeletal infections (Peleg and Munckhof 2004; Peleg et al. 2005; Nourse et al. 2007; Risson et al. 2007; Tong et al. 2008). Many of these infections occurred in young people, including children, and some were fatal.

We recently reported on the molecular epidemiology of ST93 in Australia, based on an analysis of 58 isolates collected from around Australia between 1992 and 2009 (Coombs et al. 2012). PFGE, DNA microarray, SCC*mec,* and *dru* typing were performed. These analyses confirmed the clonal nature of ST93 but were unable to shed light on the population structure and evolution of ST93. In this study, we compared whole-genome sequences of 56 of these isolates to establish a high-resolution phylogeny and evolutionary history of this emergent *S. aureus* clone and investigate molecular genetic and phenotypic variation that may explain the high virulence and success of this clone.

## Materials and Methods

### Bacterial Strains and Phenotype Measurements

The 56 *S. aureus* ST93 isolates analyzed in this study have been described previously (Coombs et al. 2012). However, alternative strain names were assigned to the isolates and these are listed in supplementary figure S1, Supplementary Material online. Isolates were grown in Heart Infusion Broth (Oxoid), incubated at 37 °C. Oxacillin susceptibility testing using Etest was performed according to manufacturer’s instructions (AB Biodisk). For δ-hemolysin (Hld) expression levels, bacteria were grown overnight at 37 °C in tryptone soy broth (TSB, Oxoid). Cultures were then diluted 1:100 into fresh media and incubation continued with shaking (180 rpm) for approximately 24 h (OD600 2.0). Culture supernatants were harvested by centrifugation and filter sterilized. These assays were performed with at least biological triplicates for each *S. aureus* isolate. Hld was measured using high-performance liquid chromatography on an Agilent Technology 1200 Series system with an analytical Agilent Eclipse XDB-C18 (4.6 mm × 150 mm) column, coupled with electrospray ionization mass spectrometry as described (Gao et al. 2013).

### Genome Sequencing and Analysis

Genomic DNA was extracted from all isolates and subjected to whole-genome shotgun sequencing using an Illumina HiSeq-2000 with 100-bp, paired-end TruSeq chemistry. Sequence reads were submitted to National Center for Biotechnology Information GenBank and are available under BioProject PRJNA232112. A read mapping approach was used to align the reads from these isolates to the *S. aureus* ST93-IV MRSA JKD6159 reference genome using SHRiMP v2.0 (Rumble et al. 2009). Those positions in the reference that were covered by at least three reads from every isolate defined a core ST93 genome. Single-nucleotide substitution polymorphisms (SNPs) and indels up to 10 bp and their predicted consequences on gene function were identified using Nesoni v0.109, a Python utility that uses the reads from each genome aligned to the core genome to construct a tally of putative differences at each nucleotide position (including substitutions, insertions, and deletions) (http://bioinformatics.net.au/, last accessed February 10, 2014) (supplementary table S1, Supplementary Material online). To define the ST93 pan genome and investigate the total *S. aureus* ST93 gene content, sequence reads for each isolate were subjected de novo assembly using Velvet v1.2.06 (Zerbino and Birney 2008), and resulting contigs were aligned to the JKD6159 genome with MUMmer (Kurtz et al. 2004). Sequences of at least 100 bp that were not present in the reference genome were extracted from contigs and appended to the JKD6159 genome to construct a pan genome sequence, which was annotated using Prokka (http://bioinformatics.net.au/, last accessed February 10, 2014). The proportion of the length of each annotated gene covered by reads was assessed for each isolate and a map summarizing all variable genes and their distribution in each strain produced (fig. 3, supplementary table S2, Supplementary Material online).

### Phylogenetics

**Fig. 1.—:**
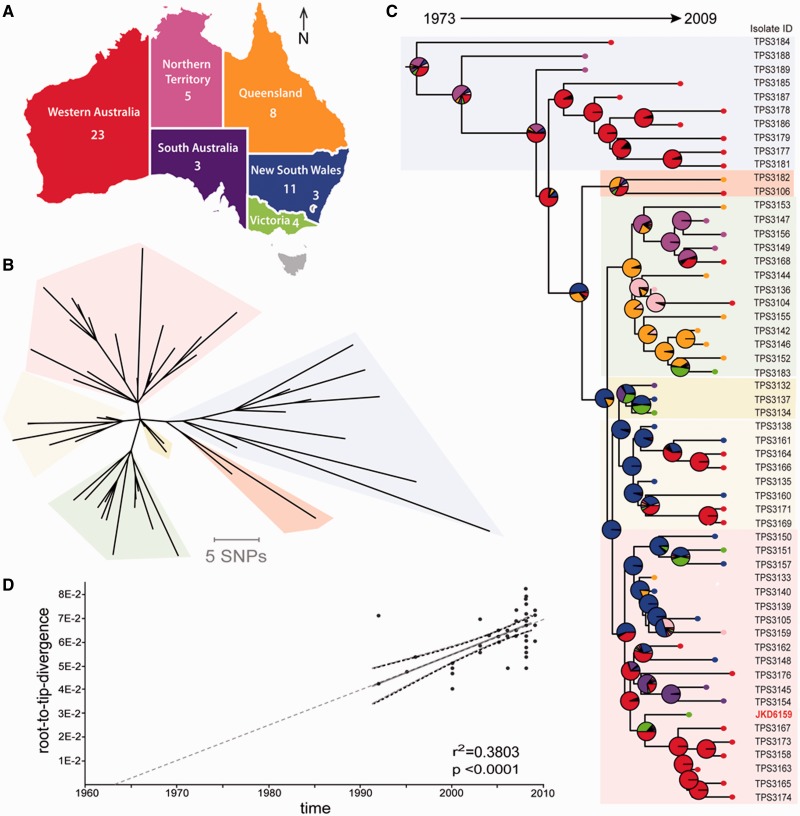
SNP-based phylogeny of ST93 *Staphylococcus aureus* based on whole-genome comparisons of 56 isolates. (*A*) Map of Australia showing the number of isolates and their origin by state or territory. The small inset within NSW indicates the Australian Capital Territory. (*B*) Unrooted neighbor-joining phylogeny showing the presence of six clades (shaded), based on alignment of 519 SNPs using uncorrected *P* distances (SplitsTree4 v 4.13.1) (Huson and Bryant 2006). (*C*) ML phylogeny based on the same SNP data with ancestral state reconstruction. The tree was rooted using *Staphylococcus epidermidis* as an outgroup, and the log likelihood estimates for location are shown in the pie charts at each node. Tips are color coded to match the state of origin. (*D*) Correlation of isolation date with ML root-to-tip branch length calculated with Path-O-Gen, showing evidence of a molecular clock-like signal in the data.

Phylogenetic analyses were undertaken using several approaches. Split decomposition and neighbor-joining analyses were performed using uncorrected *P* distances as implemented in SplitsTree4 (v 4.13.1) (Huson and Bryant 2006). The inputs for each method were the nucleotide sequence alignments of the concatenated variable nucleotide positions for the core genome among all isolates, prepared using Nesoni as described earlier and managed with SeaView v4.3.3 (Gouy et al. 2010). A phylogeny was also inferred by maximum likelihood (ML) as implemented in RAxML (Stamatakis et al. 2005), using the general time reversible (GTR) model of nucleotide substitution. Path-O-Gen was used to investigate the linear association between ML root-to-tip branch lengths and year of isolation (fig. 1) (http://tree.bio.ed.ac.uk/software/pathogen/, last accessed February 10, 2014). BEAST v1.7.4 (Drummond and Rambaut 2007) was used to infer the evolutionary dynamics of ST93 *S. aureus* with a GTR + Γ nucleotide substitution model and tip dates defined as the year of isolation. Multiple analyses were run with both constant population size and Bayesian skyline demographic models, in combination with either a strict molecular clock or a relaxed clock with uncorrelated lognormal distribution. Both demographic models, using lognormal or strict clock, yielded almost identical results. For sampling the posterior probability distributions and analyses of all model combinations (demographic and clock), ten Markov chain Monte Carlo (MCMC) chains of 100 million generations each were run to ensure convergence, with samples taken every 1,000 MCMC generations. Replicate analyses were combined and parameter estimates calculated with Tracer v1.5 (Drummond and Rambaut 2007).

For phylogeographic analysis, location (the state within Australia from which the isolate was obtained) was treated as a discrete character trait, and ML ancestral reconstruction was used to estimate the likely location of unsampled, ancestral forms of ST93. The same results were obtained using alternative implementations of ML ancestral reconstruction in *R*—the ace function in the ape package (plotted as pie graphs in fig. 1*C*) and pml functions in the phangorn package.

**Fig. 2.—:**
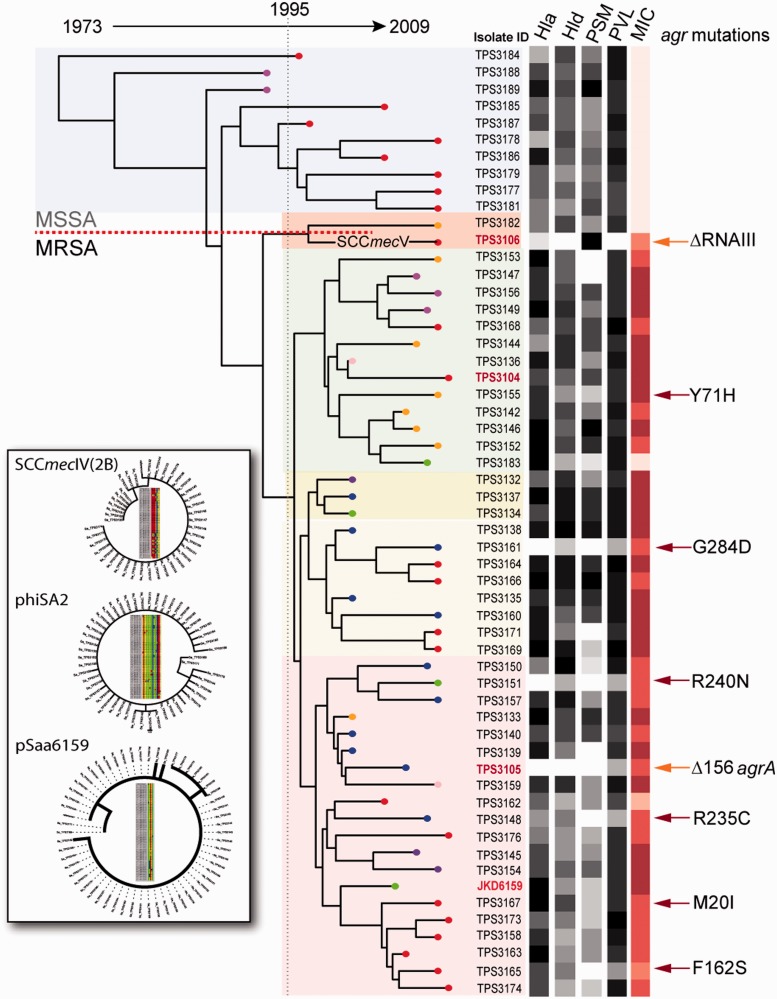
ML phylogeny of *Staphylococcus aureus* ST93. Shading indicates the six phlyogenetically distinct clusters. Dating of nodes is derived from BEAST analysis and shows acquisitions of SCC*mec* elements around 1995. All nodes had more than 90% bootstrap support. Phenotype data for expression of four exotoxins (Hla; Hld; PSM; phenol soluble modulin α3; and PVL, panton valentine leukocidin) and oxacillin MIC displayed by heatmap and aligned with tree tips. Depicted also are the positions of *agrC* mutations (red arrows) and two other *agr* mutations (orange arrows: deletion of RNAIII for TPS3106 and deletion in *agrA* for TPS3105), aligned with their corresponding taxa. Inset shows neighbor-joining phylogeny derived from independent alignment of the sequences of three key accessory elements, with tree topologies supporting acquisition and coevolution of these elements with the ST93 MRCA. A high-resolution version of this inset figure is provided in supplementary figure S3, Supplementary Material online.

Previously established mean exotoxin expression levels for Hla, and PSMα3 (Chua KYL, Monk IR, Lin Y, Seemann T, Tuck KL, Porter JL, Stepnell J, Coombs GW, Davies JK, Stinear TP, Howden BP, submitted for publication), together with Hld expression levels and oxacillin MICs determined in this study were visualized alongside the ML phylogeny (fig. 2). The mean expression levels based on biological triplicate measurements were also compared between pairs of strains using a two-sided, unpaired *t-*test on log_10_ transformed exotoxin expression values (GraphPad Prism V6). The null hypothesis (no difference between means) was rejected for *P* < 0.05. For each MRSA isolate, mean exotoxin expression and oxacillin MIC were compared using the nonparametric Spearman correlation analysis (GraphPad Prism V6).

For correlation of phenotypes with phylogenies, Felsenstein's phylogenetically independent contrasts (PIC) method was employed (Felsenstein 2008), and the PIC test was performed using the R+ package “ape” (http://ape-package.ird.fr/, last accessed February 10, 2014). Conventional correlation tests assume data points (here, strains) are independent, which is not the case for phylogenetically related strains, as closely related strains may be expected to be more alike in phenotype than distantly related strains. The PIC method takes this into account by instead assuming that variation in an observed variable is due to Brownian motion along the branches in the phylogenetic tree (Felsenstein 2008).

## Results

### Defining a *S. aureus* ST93 Core Genome and Phylogeny

Alignment of sequence reads from the 56 isolates to the *S. aureus* JKD6159 reference genome defined a 2,625,004 bp core genome (93.4% of the 2,811,434 bp JKD6159 genome). Comparisons of this core genome among all isolates identified only 519 SNPs, confirming limited genetic diversity within this clone. An alignment of the concatenated variant nucleotides was then used for phylogenetic analyses. Split decomposition analysis produced a tree with six distinct clades and a nonnetworked topology, suggesting recombination was not significant among this population of isolates (fig. 1*B*). A rooted phylogeny was also inferred by ML, using *Staphylococcus epiderimidis* R6P2A as the outgroup, and this produced a robust tree with substantial bootstrap support that also resolved into the same six clades as split decomposition (fig. 1*B* and *C*). There was a significant positive correlation between ML root-to-tip branch lengths and dates of isolation, suggesting substitution mutations have been occurring at a regular rate (fig. 1*D*). There was significant geographical clustering of isolates within the phylogeny, with most states being represented in just two phylogenetic clades (fig. 1*C*). However, isolates from WA were distributed throughout the tree, in five of the six clades, separated by deep branching (fig. 1*C*). Thus, most of the diversity identified among ST93 was present in WA, where the clone was first identified. This suggests that ST93 diversified in WA early on before spreading out into other states, and ML ancestral reconstruction (pie charts in fig. 1*C*) supports this hypothesis. The deep branching of Northern Territory isolates, and their early detection in the late 1990s, suggests the origin of ST93 may lie in the north west of Australia. In contrast, isolates from QLD clustered quite tightly, making QLD an unlikely origin for ST93. However, the isolate collection is somewhat dominated by relatively recent strains from 2008 and contains more isolates from WA than from other states (fig. 1*C*), lending some caution to this interpretation.

### Estimating *S. aureus* ST93 Mutation Rate

We used BEAST to assess the population structure and evolutionary dynamics of *S. aureus* ST93, including estimating mutation rates and divergence times as shown in supplementary figure S2, Supplementary Material online. The divergence time for the most recent common ancestor of this collection of isolates was 1973 (37 years prior to 2009, 95% highest posterior density [HPD]: 1963–1984), and the mutation rate (SNP/year) across the alignment was 2.3 × 10^−^^3^. Scaled to the proportion of sites, this represents within the core genome, the approximate rate of SNP/site/year is estimated at 4.5 × 10^−^^7^ (95% HPD: 3.8 × 10^−^^7^ to 5.5 × 10^−^^7^). SNPs were also mapped back to the BEAST-inferred phylogeny to look for homoplasies, but none were detected.

### Acquisition of SCCmec and Key Accessory Elements

Two SCC*mec* types are represented in this collection. Isolate TPS3106 harbors SCC*mec*V[5C2&5], whereas the other 43 MRSA harbor SCC*mec*IV[2B]. Alignment of the 22,168 bp SCC*mec* IV[2B] sequence among the 43 isolates identified only eight SNPs. This was insufficient variation to infer a very informative phylogeny, but two of the MRSA clades identified by whole-chromosome SNP alignments similarly clustered in the tree inferred from the SCC*mec*IV[2B] SNP alignments (fig. 2, supplementary fig. S3, Supplementary Material online). A single acquisition of SCC*mec*IV[2B] is the most parsimonious explanation for this pattern, with BEAST analysis indicating that the MRCA of ST93 MRSA emerged around 1995, as shown in figure 2 and supplementary figure S2, Supplementary Material online.

All 56 isolates contained the previously described 21 kb MW2-like plasmid (Chua et al. 2011). Alignment of the plasmid sequences for each isolate identified only five variable positions, consistent with the plasmid coevolving with the chromosome among the ST93 population as shown in supplementary figure S3, Supplementary Material online. Similarly, the 46-kb phiSA2 prophage harboring the *pvl* genes was present in all isolates (except TPS3105 and TPS3161) and sequence alignments displayed only 18 variable positions (45,768 bp), suggesting that this prophage was present in the MRCA of these isolates and has coevolved with the chromosome, summarized in supplementary figure S3, Supplementary Material online.

### Linking Changes in Exotoxin Expression with Phylogeny and Genotype

A hallmark of the reference strain JKD6159 is its high virulence, linked to its robust expression of Hla (Chua et al. submitted). We were interested to explore the differential virulence potential of the 56 isolates and to compare virulence differences with potentially causative genetic differences. We have previously established by quantitative Western immunoblotting (Chua KYL, Monk IR, Lin Y, Seemann T, Tuck KL, Porter JL, Stepnell J, Coombs GW, Davies JK, Stinear TP, Howden BP, submitted for publication) that the majority of ST93 isolates produced substantial levels of Hla that were not significantly different from each other but were above that produced by *S. aureus* USA300, whereas a handful of ST93 isolates displayed reduced expression. In this study, genome analysis revealed that four isolates with low expression levels (TPS3105, TPS3106, TPS3151, and TPS3161) were phylogenetically distant from one another, but the low expression of each isolate could explained by an independent mutation affecting the *agr* locus (fig. 2). The *agr*-encoded regulator is the major controller of Hla expression, as repair of an *agrA* frame-shift mutation in TPS3105 restored expression levels and strain virulence to that of JKD6159 (Chua KYL, Monk IR, Lin Y, Seemann T, Tuck KL, Porter JL, Stepnell J, Coombs GW, Davies JK, Stinear TP, Howden BP, submitted for publication). Other than the deletion of RNAIII in TPS3106, the seven other *agr* mutants had predicted amino acid substitutions in AgrC*,* although only three *agrC* mutations were linked to reduced Hla expression (fig. 2). None of the MSSA isolates displayed sequence variations in *agr* (fig. 2). As previously reported, PVL expression remained constant and high among all isolates, with reduced PVL expression co-occurring with mutations in the *agr* locus (fig. 2) (Coombs et al. 2012).

We also attempted to explore the links between genotype and expression of the exotoxins PSMα-3 (previously measured) and Hld. Low exotoxin expression was sometimes (but not always) associated with *agr* mutations. TPS3106 demonstrated relatively high levels of PSM production, despite a defective *agr* (fig. 2). There were also instances of the inverse, where low Hld or PSMα-3 expression occurred in the absence of any *agr* mutations (TPS3139, TPS3153, TPS3171, and TPS3183), indicating that other regulatory systems also control expression of these genes.

### Whole-Genome Comparisons of Closely Related Strains with Divergent Exotoxin Expression

To try and ascertain the genetic basis for *agr*-independent “exotoxin-low” phenotype among these four strains, we looked for mutations within (or around) other virulence gene regulators including *rot*, *sarR*, and *saeRS* relative to the reference genome JKD6159, but no changes were found as listed in supplementary table S1, Supplementary Material online. We then compared phylogenetically matched pairs for the four isolates, where the partner strain had significantly higher exotoxin expression, to exploit their close genetic relationship and thus attempt to identify additional regulatory controls of virulence. The findings are summarized in table 1 and provided in full detail in supplementary table S3, Supplementary Material online. There was a diverse array of genome changes for each pair, with no two pairs sharing mutations in the same genes, suggesting there are multiple pathways available to *S. aureus* for additional modulation of exotoxin production. For TPS3139/3140 and TPS3171/3161, there were less than ten genetic changes within each pair. These differences included mutations in genes encoding hypothetical proteins (or their putative promoter regions), including a predicted transcriptional regulator (SAA6159_00471) in TPS3171. These genes would be candidates for future functional studies to explore their role in control of toxin expression. The other two isolate pairs were more distant from each other and had a larger number of SNPs. Among the differences between these isolates were predicted mutations in genes encoding RpoB (A666V), and FmtC (V205G) for TPS3183, proteins known to alter virulence and antibiotic susceptibility (Komatsuzawa et al. 2001; Gao et al. 2013), although these were both quite conservative amino acid substitutions (table 1). TPS3183 has also lost SCC*mec.*

**Table 1:** Genetically Matched Pairs with δ-Toxin or PSMα3 Expression Differences

Pair (Expression)	Strain	Expression (µg/ml, Mean ± SD)		Core Genome Changes	Pan Genome Changes	Comments
		δ-Toxin	PSMα3^a^			
1 (low)	TPS3139	10.21 ± 0.17^b^	ND^c^	7 SNPs (2 nonsynonymous)	None	One intergenic SNP u/s of SAA6159_02065, encoding a PTS system mannitol-specific IIBC component.
1 (high)	TPS3140	12.67 ± 0.41	24.37 ± 1.09			
2 (low)	TPS3147	10.78 ± 0.30	3.28 ± 0.93	21 SNPs, 3 InDels (3 nonsynonymous)	None	Two loss-of-function mutations include a frame-shift in SAA6159_00741, encoding a hypothetical secreted protein, and a stop codon in a putative ATPase, SAA6159_02306.
2 (high)	TPS3156	13.36 ± 0.66	25.31 ± 2.01			
3 (low)	TPS3171	9.71 ± 1.67	ND	5 SNPs	None	Substitution u/s of SAA6159_00471, of a putative GntR family transcriptional regulator; potential regulatory region for this CDS.
3 (high)	TPS3169	16.08 ± 0.32	9.89 ± 0.46			
4 (low)	TPS3183	5.17 ± 0.76	3.67 ± 0.41	40 SNPs, 4 InDels	Loss of SCCmec in TPS3183	Nonsynonymous SNPs include RpoB (A666V), nitrate reductase beta subunit (F427Y) and oxacillin resistance protein FmtC (V205G).
4 (high)	TPS3152	13.31 ± 0.83	29.79 ± 4.94			

^a^Formylated peptide.

^b^All expression values were significantly different (*P* < 0.01) except for δ-toxin expression between 3,139 and 3,140.

^c^Not detected.

### Oxacillin MIC and Exotoxin Expression Are Positively Correlated in ST93

**Fig. 3.—:**
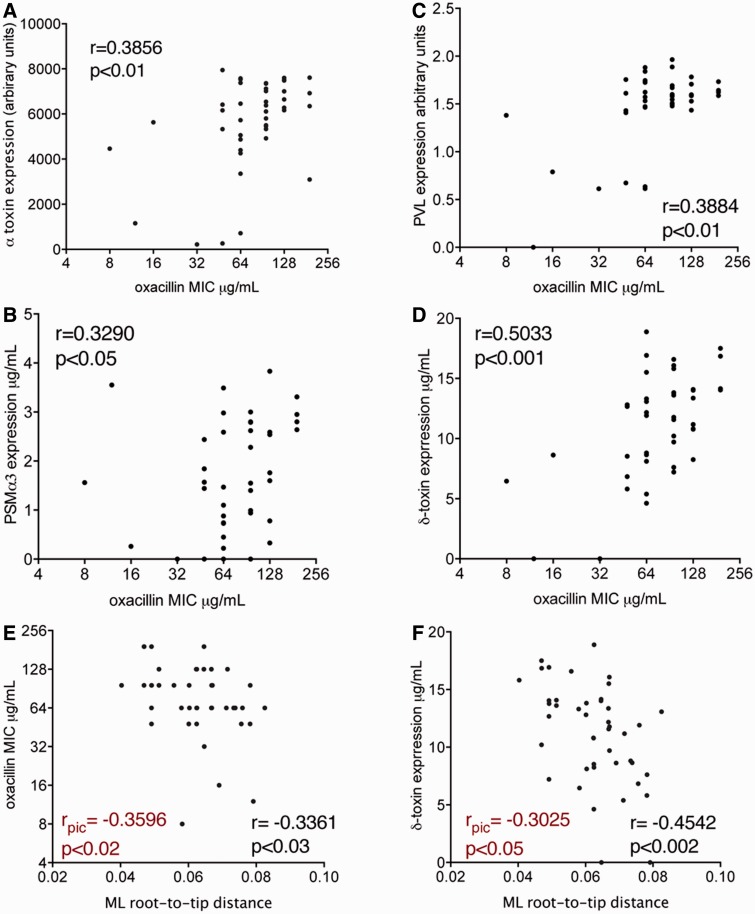
Exotoxin expression among 43 ST93 MRSA isolates compared with oxacillin MIC and evolutionary distance. Correlation of (*A*) Hla, (*B*) PSMα3, (*C*) Hld, and (*D*) PVL expression with oxacillin MIC. Shown is Spearman's rank correlation coefficient (*r*) and significance indicated with a two-sided *P* value. Correlation of ML root-to-tip distance with (*E*) oxacillin MIC and (*F*) Hld are also shown. Assessment of the significance of these relationships was also measured using Felsenstein’s PIC method (Felsenstein 2008). The correlation coefficient of this statistic is indicated by *r*_pic_.

Several recent studies have observed a link between expression of the *mecA*-encoded penicillin-binding protein PBP2a and *agr*-controlled toxin expression, with higher levels of PBP2a associated with reduced toxin expression through interference with *agr* quorum sensing (Rudkin et al. 2012, 2014). SCC*mec*IV[2B] (as present in ST93 and other CA-MRSA clones) has been linked to lower PBP2a expression, leading to higher toxin expression (Rudkin et al. 2014). To explore this phenomenon in ST93, we measured the oxacillin MIC for all 56 isolates and compared these values with our exotoxin measurements for the same isolates (figs. 2 and 3*A*–*D*). Contrary to expectations, where a higher MIC would predict more PBP2a, leading to reduced *agr* activity and therefore reduced toxin expression, a significant positive correlation was actually observed between all four exotoxins and MIC, suggesting an inverse relationship between *agr* and SCC*mec*IV[2B]. This relationship was most clearly evident between Hld and MIC (fig. 3*D*).

### Decreasing Oxacillin MIC and Exotoxin Expression Over Time

We also examined the change in oxacillin MIC over the evolution of this ST93 strain collection. A modest but significant correlation was observed between MIC and ML phylogeny root-to-tip branch length, suggesting that MICs are decreasing as the complex evolves (fig. 3*E*). This relationship is visually evident in the alignment of mean MIC values, displayed as a heatmap and aligned with the ML phylogeny as shown in figure 2. A similar, significant relationship was also observed between Hld expression and branch length (figs. 2 and 3*F*), underscoring the link between SCC*mec* and *agr*-regulated virulence gene expression, and suggesting that this clone is undergoing adaptive change.

### ST93 Pan-Genome Analysis

**Fig. 4.—:**
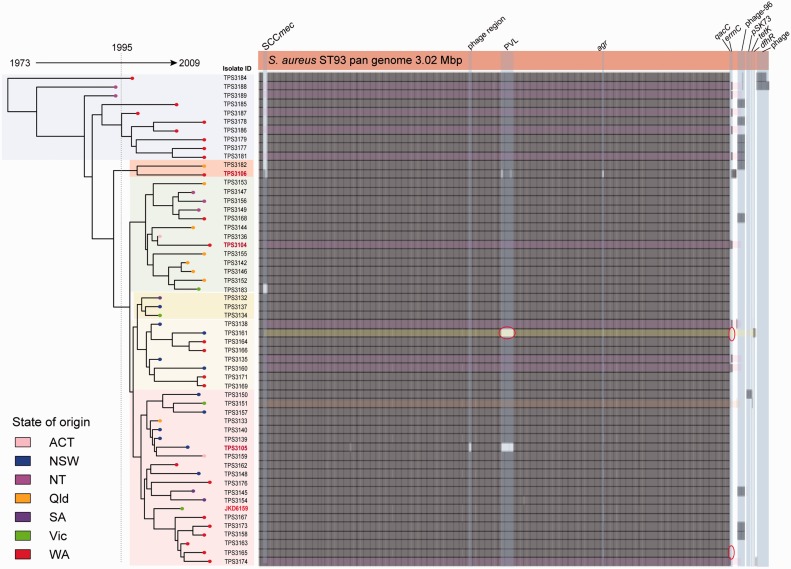
The *Staphylococcus aureus* ST93 pan genome aligned with the ML phylogeny. The upper orange block represents the length of the pan genome. Shown in each row of gray shading is the proportion of the pan genome present in each isolate. Colored shading highlights the presence of selected antibiotic resistance elements (pink: erythromycin; yellow: trimethoprim; orange: tetracycline). Red encircled areas highlight discrepancies for one isolate (TPS3161) with previous DNA microarray data (Coombs et al. 2012).

Analysis of the 3.02 Mb ST93 pan genome showed only limited variability among the 56 isolates as depicted in figure 4, supplementary figure S1 and table S2, Supplementary Material online, again consistent with the recent dissemination of ST93. However, several important accessory elements were variously present, including two prophage, one resembling phage96 (ST93P_02827–ST93P_02903) (Kwan et al. 2005), and ten occurrences of another large phage of unknown function (ST93P_02719–ST93P_02767). Key features were the presence in ten isolates of a pNE131-like plasmid harboring the *ermC* gene (ST93P_02678), encoding erythromycin resistance (Lampson and Parisi 1986). The distribution of this plasmid among isolates dispersed across the core genome phylogeny suggests this plasmid has been acquired on several occasions. Other independent gene acquisitions include *dfhR* (STP93_02815) conferring trimethoprim resistance (Rouch et al. 1989), *tetK* (STP93_02803) encoding tetracycline resistance (Khan and Novick 1983), and three instances of *qacC* (STP93_02677), responsible for increased tolerance to quaternary ammonium compounds and beta-lactam antibiotics (Fuentes et al. 2005).

The pan genome data aligned well with previous PFGE and DNA microarray analysis (Coombs et al. 2012). However, there were also some differences as shown in supplementary figure S1, Supplementary Material online. Accessory genome variations as revealed by the pan genome analysis, in particular the different phage content, likely explains the different PFGE types previously described for this clone (fig. 4). These data are described in more detail in supplementary figure 1 and table S2, Supplementary Material online.

## Discussion

The emergence of CA-MRSA as an important pathogen has occurred over the past 2 decades (David and Daum 2010; DeLeo et al. 2010). Although much attention has focused on the USA300 epidemic in the Unites States (Uhlemann et al. 2013), globally a number of other important but genetically distinct clones of CA-MRSA have been reported (Chua, Laurent, et al. 2011). A recent study described the population structure and evolutionary history of the emerging CA-MRSA clonal complex 97, and the evidence for several bovine-to-human adaptive leaps within this lineage of *S. aureus* (Spoor et al. 2013). Identifying drivers of CA-MRSA evolution, such as human–livestock interactions will be key to understanding and attempting to control the spread of new *S. aureus* clones (Shepheard et al. 2013). In Australia, a geographically isolated continent, ST93 has become the dominant clone of CA-MRSA, with clinical and epidemiological features reminiscent of early reports of the USA300 epidemic in the Unites States, including rapid emergence and epidemiological dominance in addition to severe clinical manifestations (Peleg and Munckhof 2004; Peleg et al. 2005; Chua et al. 2011; AGAR 2012; Coombs et al. 2012). This has led to concern regarding the origin of ST93 *S. aureus*, including the dynamics of acquired methicillin resistance, and the virulence potential of the ST93 population in Australia. *Staphylococcus aureus* is known to cause clonal epidemic waves of infection, such as that caused by phage type 80/81 in the 1950s (DeLeo et al. 2011), and detailed comparative genomics is starting to provide important insights into the molecular basis of *S. aureus* virulence and the epidemiology of *S. aureus* infections (Kennedy et al. 2008; DeLeo et al. 2011; McAdam et al. 2012). Here, we have used genomics to enhance our understanding of the population structure and evolution of ST93 CA-MRSA from Australia, providing new insights into the emergence of this clone. We found limited DNA sequence diversity despite the geographic distribution of our sample collection, suggesting that ST93 is a recently emerged clone. In fact, the total number of SNPs in the core genome (2.6 Mb) of our 56 ST93 isolates was restricted to 519. Using alignments of core genome SNPs and analysis with BEAST, we estimated a mutation rate that is 10–20 times less than rates previously reported for other *S. aureus* clones (Harris et al. 2010; Nubel et al. 2010; Kurt et al. 2013). The significance of this difference is not known and may represent a clone-specific phenomenon. Our phylogeographic analysis indicates the origins of present-day ST93 CA-MRSA around Australia was a single-methicillin-sensitive, PVL-positive ST93 *S. aureus* ancestor, which emerged in North WA in the 1960s. This is consistent with the earliest records of ST93 *S. aureus*, which was first reported in this region in 1993 (Udo et al. 1993), followed by later reports in QLD (Munckhof et al. 2003) and other states (Nimmo et al. 2000; Gosbell et al. 2001).

Previously, we fully sequenced and finished a highly virulent ST93 strain, JKD6159 (Chua et al. 2011). Despite the substantial genetic diversity of this clone compared with other fully sequenced *S. aureus*, very few clone-specific coding sequences were identified. We investigated the accessory elements of the whole ST93 *S. aureus* population using a pan genome analysis of all isolates in this study (fig. 4). The pan genome was 3 Mb and comprised additional mobile DNA elements typically identified in *S. aureus*, harboring resistance elements to erythromycin (*ermC*), trimethoprim (*dfhR*), and tetracycline (*tetK*). Erythromycin resistance was independently acquired by multiple MSSA and MRSA strains, whereas *tetK* was restricted to one strain (TPS3151) and *dfhR* to one strain (TPS3161). Characterization of the SCC*mec* element identified two unique *mec* types, with phylogeny of the *mec* elements indicating two unique SCC*mec* acquisitions in the study population, followed by clonal expansion of MRSA, predominately containing SCC*mec* IV[2B]. Repeated SCC*mec* acquisition cannot be ruled out, as has been previously suggested in this clone (Tong et al. 2009), but this seems less likely. Loss of PVL phage was observed for strains TPS3161 and TPS3105, and loss of SCC*mec* for strain TPS3183.

In this study, we also investigated the virulence characteristics of the isolate collection by measuring exotoxin expression, including PVL, Hla, Hld, and PSMα3. Our reference ST93 CA-MRSA strain JKD6159 has been shown to be the most virulent global isolate of *S. aureus* tested to date in both skin and sepsis models (Chua et al. 2011; Tong et al. 2013), therefore understanding the genomic mediators of virulence in this clone is likely to uncover important features of *S. aureus* virulence. A large number of distinct CA-MRSA clones have now been fully sequenced, and to date, no single gene or locus has been discovered to account for the enhanced virulence of these clones compared with hospital type MRSA. In fact, rather than enhanced virulence being explained by the addition of virulence factors, virulent CA-MRSA may represent “cleanskin” *S. aureus* that has been relatively unexposed and unaffected by the selective pressures of antibiotics and the hospital environment (Uhlemann et al. 2013) and has therefore not acquired virulence tempering mutations. It has been clearly demonstrated that accumulation of *agr* mutations is associated with MRSA adaptation to the hospital environment (Shopsin et al. 2008; Fitzgerald 2013), possibly as an adaptive response to antibiotic exposure (Paulander et al. 2013). Additionally, in the CC30 clone, it appears that mutations in *hla*, *agrC,* and possibly *crtM* (squalene desaturase) are associated with the loss of virulence and evolution toward a healthcare-associated clone with epidemic potential (DeLeo et al. 2011; McAdam et al. 2012). On the other hand, recent epidemic clones, such as ST22, are also highly successful and do not harbor these mutations (Holden et al. 2013). Although ST93 is currently recognized as a community MRSA clone, using analysis of exotoxin expression, we have detected an evolutionary trend toward reduced virulence and lower MIC, and the acquisition of eight independent *agr* mutations in the 56 isolates from this study may indicate adaptation to health care environments. The virulence analysis highlights a number of important features. First, despite the relative genetic homogeneity of the isolate collection, significant differences in exotoxin expression were also observed, suggesting that the potential to cause severe clinical disease would be different in these isolates. So, although generalizations about the virulence characteristics of a particular clone of *S. aureus* can be made, individual isolates within an MLST-defined clonal group can behave very differently, and these differences are not solely explained by *agr* mutations. Future studies investigating the virulence characteristics of a clone need to keep this in mind and present data from a number of representative isolates. In addition, although JKD6159 has been most extensively studied and found to be distinctive in its high virulence capacity, many other strains from this collection produced similar amounts of Hla to JKD6159 but significantly more PSMα3, as shown in figure 2 (TPS3138, TPS3146, and TPS3166), possibly indicating they could be even more virulent.

The high virulence potential of individual clones should also be considered in the context of the ST93 population, where there was a significant trend toward reduced *agr* activity, as indicated by the correlation between evolutionary recent isolates and reduced Hld expression (figs. 2 and 3*F*). These data suggest that the ST93 population is under pressure to change. Reduced virulence over time is typical of hospital-associated MRSA and is thought to reflect adaptation to health care settings (DeLeo et al. 2011). Perhaps, the presence of this phenotype in ST93 CA-MRSA indicates reduction in virulence is a more generalizable trait of MRSA, where acquisition of SCC*mec* leads to alteration of *agr* function (and perhaps other regulators) and subsequent adaptive changes to obtain a regulatory homeostasis, balancing the impact of carrying SCC*mec* on gene regulation with bacterial cell functions required for persistence in human populations. The significant positive correlations between expression of exotoxins and oxacillin MIC are consistent with an interaction between *agr* and SCC*mec* (fig. 3*A*–*D*). Recent studies have reported a link between oxacillin MIC and *agr*, where PBP2a (*mecA*)-induced cell wall changes are proposed to interfere with the ability of the *agr* system to sense autoinducing peptide (Rudkin et al. 2012, 2014). This model predicts that *agr* activity will increase as oxacillin MIC decreases. However, this is the inverse relationship to the positive correlation between oxacillin MIC and *agr* function (using Hld production as a marker of *agr* activity) that we have observed (fig. 3*D*). Future research measuring RNAIII and *mecA* expression levels among our ST93 strain collection might provide a more direct exploration of this relationship.

The ST93 population structure uncovered from this strain collection suggested region specific clonal expansion (fig. 1), indicative of the establishment of localized populations and local transmission. However, significant ongoing country-wide mixing also seems to be occurring, reminiscent of the pattern of *Enterococcus faecium* movement around Australia (Howden et al. 2013). Investigating the movement of ST93 CA-MRSA could be more comprehensively addressed with a larger isolate collection that is prospectively collected and supported with detailed microbiological and epidemiological data.

## Supplementary Material

Supplementary tables S1–S3 and figures S1–S3 are available at *Genome Biology and Evolution* online (http://www.gbe.oxfordjournals.org/).

Supplementary Data
