# Supplementary material for: Adaptive Change Inferred from Genomic Population Analysis of the ST93 Epidemic Clone of Community-Associated Methicillin-Resistant Staphylococcus aureus
Source: Genome Biol Evol. 2014 Jan 29;6(2):366–78. doi: 10.1093/gbe/evu022 (PMC3942038; doi:10.1093/gbe/evu022)
Supplement: Supplementary Data [file supp_6_2_366__index.html]

Adaptive Change Inferred from Genomic Population Analysis of the ST93 Epidemic Clone of Community-Associated Methicillin Resistant Staphylococcus aureus — Adaptive Change Inferred from Genomic Population Analysis of the ST93 Epidemic Clone of Community-Associated Methicillin-Resistant Staphylococcus aureus — Supplementary Data 

# Adaptive Change Inferred from Genomic Population Analysis of the ST93 Epidemic Clone of Community-Associated Methicillin-Resistant *Staphylococcus aureus*

## Supplementary Data

files

**Files in this Data Supplement:**

- Supplementary Data - zip file
