# Supplementary figures and images for: Adaptive Change Inferred from Genomic Population Analysis of the ST93 Epidemic Clone of Community-Associated Methicillin-Resistant Staphylococcus aureus
Source: Genome Biol Evol. 2014 Jan 29;6(2):366–78. doi: 10.1093/gbe/evu022 (PMC3942038; doi:10.1093/gbe/evu022)

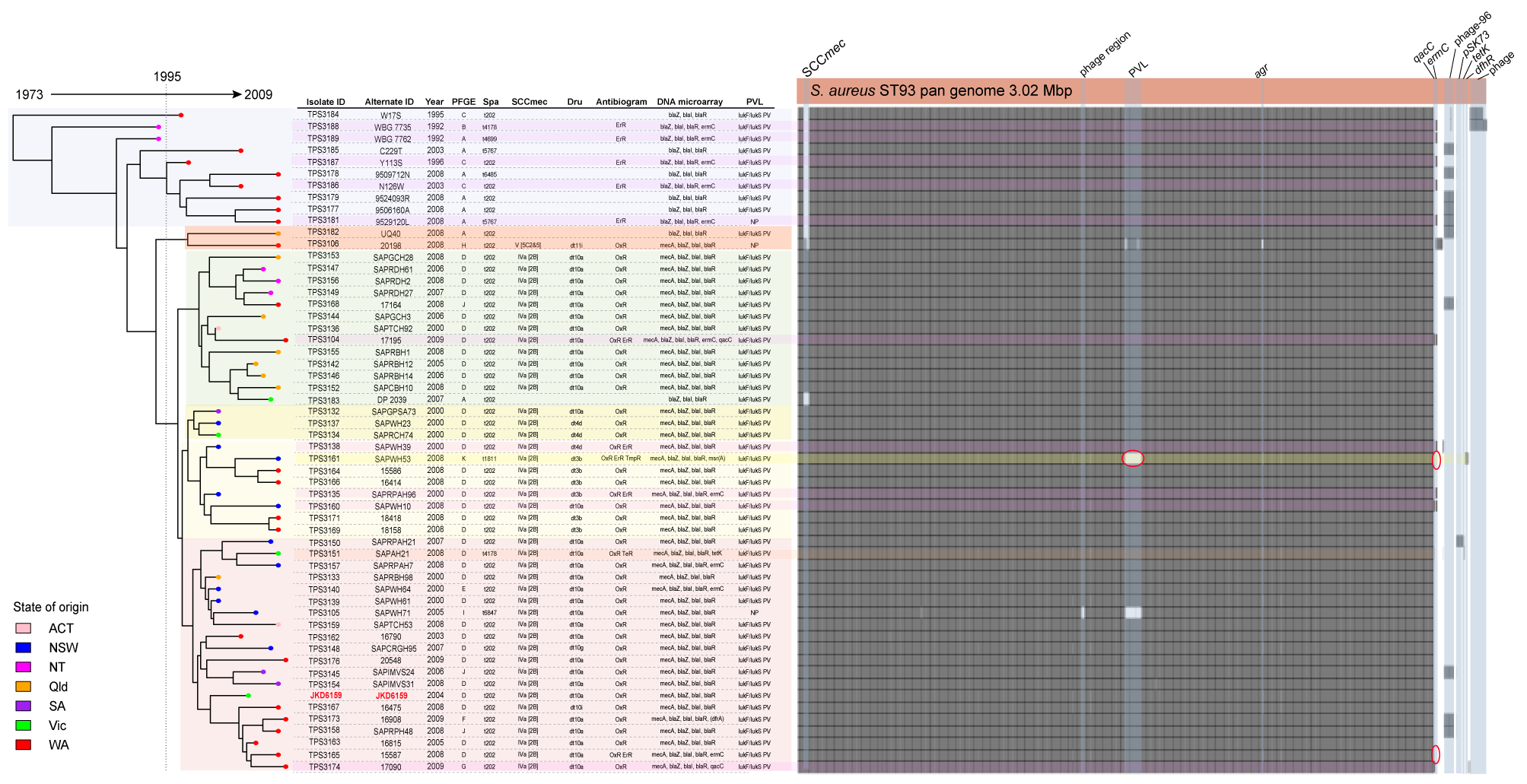

Supplement: Supplementary Data [file supp_evu022_suppl_data.zip › Fig_S1.tif]

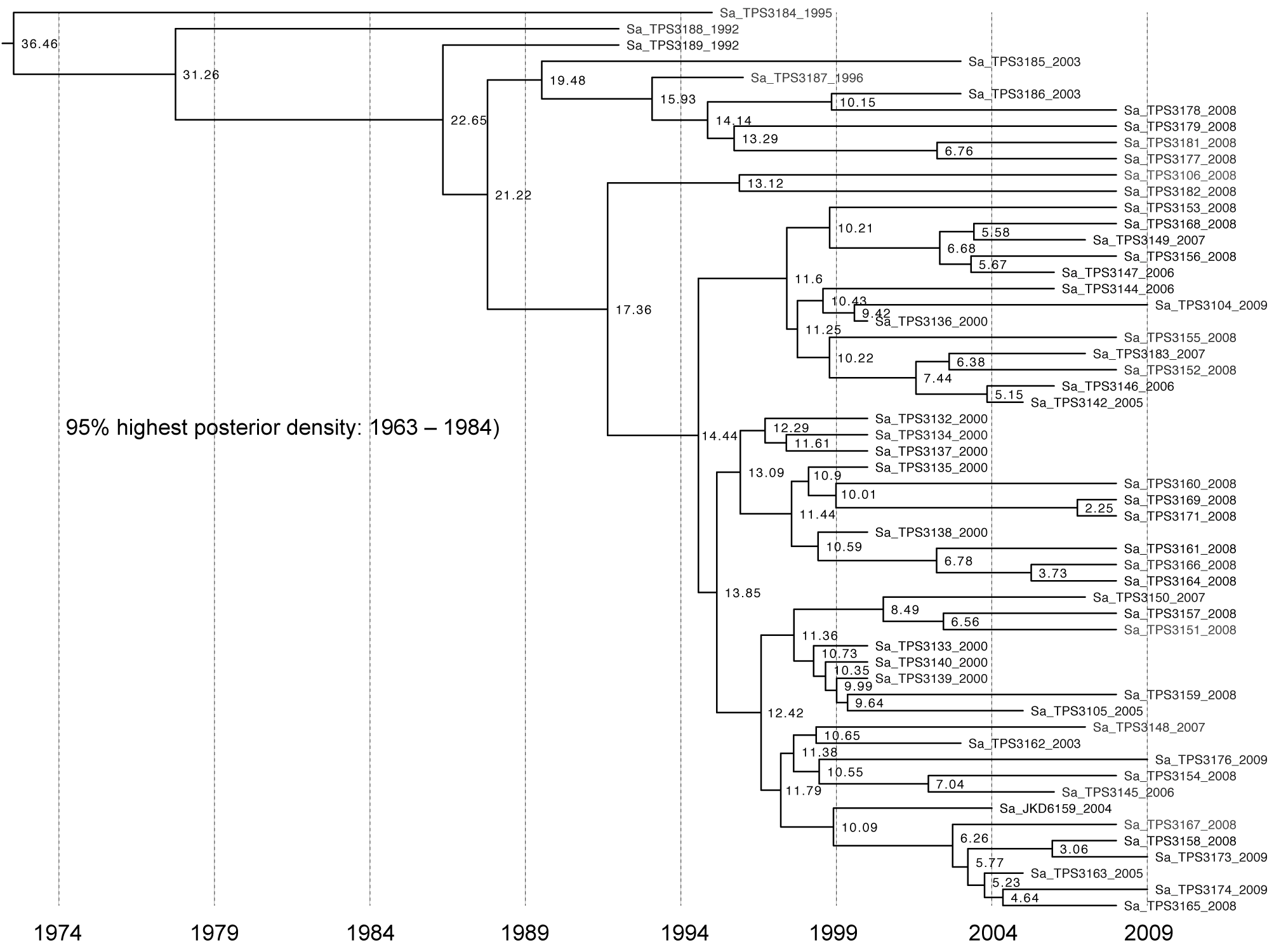

Supplement: Supplementary Data [file supp_evu022_suppl_data.zip › Fig_S2.tif]

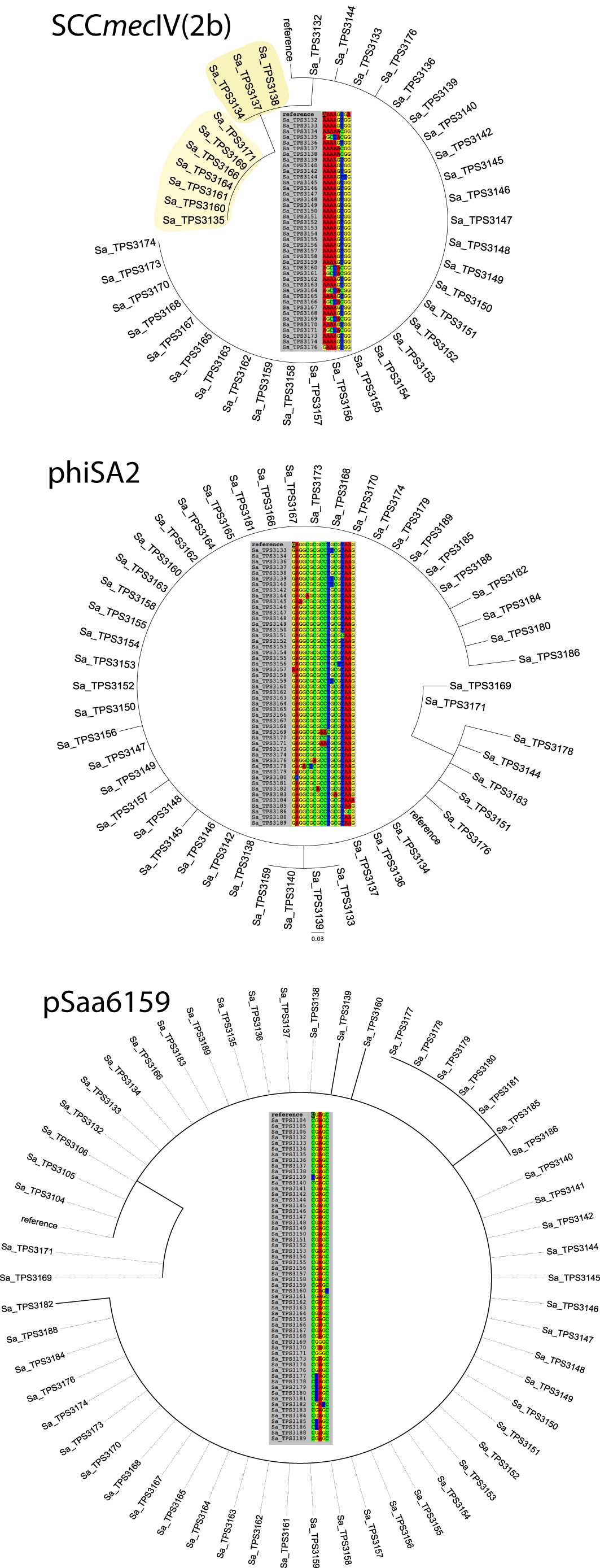

Supplement: Supplementary Data [file supp_evu022_suppl_data.zip › Fig_S3.tif]
